# Nonobese mice with nonalcoholic steatohepatitis fed on a choline‐deficient, l‐amino acid‐defined, high‐fat diet exhibit alterations in signaling pathways

**DOI:** 10.1002/2211-5463.13272

**Published:** 2021-09-21

**Authors:** Noriko Suzuki‐Kemuriyama, Akari Abe, Sae Nakane, Kinuko Uno, Shuji Ogawa, Atsushi Watanabe, Ryuhei Sano, Megumi Yuki, Katsuhiro Miyajima, Dai Nakae

**Affiliations:** ^1^ Department of Nutritional Science and Food Safety Faculty of Applied Bioscience Tokyo University of Agriculture Setagaya Japan; ^2^ Department of Nutritional Science and Food Safety Graduate School of Applied Bioscience Tokyo University of Agriculture Setagaya Japan; ^3^ Department of Food and Nutritional Science Graduate School of Applied Bioscience Tokyo University of Agriculture Setagaya Japan

**Keywords:** nonalcoholic steatohepatitis, ‘nonobese’ NASH subtype, Rho GTPases signaling

## Abstract

Nonalcoholic steatohepatitis (NASH) is often associated with obesity, but some patients develop NASH without obesity. The physiological processes by which nonobese patients develop NASH and cirrhosis have not yet been determined. Here, we analyzed the effects of dietary methionine content on NASH induced in mice fed on a choline‐deficient, methionine‐lowered, l‐amino acid‐defined high‐fat diet (CDAHFD). CDAHFD with insufficient methionine induced insulin sensitivity and enhanced NASH pathology, but without obesity. In contrast, CDAHFD with sufficient methionine induced steatosis, and unlike CDAHFD with insufficient methionine, also induced obesity and insulin resistance. Gene profile analysis revealed that the disease severity in CDAHFD may partially be due to upregulation of the Rho family GTPases pathway and mitochondrial and nuclear receptor signal dysfunction. The signaling factors/pathways detected in this study may assist in future study of NASH regulation, especially its ‘nonobese’ subtype.

## Graphical abstract

Dietary insufficiency of methionine induced nonalcoholic steatohepatitis (NASH) in mice fed on a choline‐deficient, l‐amino acid‐defined high‐fat diet, but without obesity. The upregulation of the Rho family GTPases pathway, and mitochondrial and nuclear receptor signal dysfunction may be involved. The signaling factors/pathways detected in this study may assist in future study of NASH regulation, especially its ‘nonobese’ subtype.

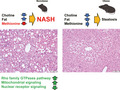

**Abbreviations**
CCR‐2: C‐C motif chemokine receptor 2
CDAHFD: choline‐deficient, methionine‐lowered, l‐amino acid‐defined high‐fat diet
GTTs: glucose tolerance tests
ITTs: insulin tolerance tests
NAFL: nonalcoholic fatty liver
NAFLD: nonalcoholic fatty liver disease
NASH: nonalcoholic steatohepatitis
PCA: principal component analysis
PPAR: peroxisome proliferator‐activated receptor
TNF‐α: tumor necrosis factor‐α
VLDL: very low‐density lipoprotein

Nonalcoholic fatty liver disease (NAFLD), a common and chronic liver condition, affects numerous individuals worldwide. NAFLD is considered a hepatic manifestation of metabolic syndrome, which constellates metabolic abnormalities, such as obesity, dyslipidemia, and insulin resistance [1]. Hepatic phenotypes of NAFLD are diverse, ranging from mild steatosis to various degrees of inflammation and fibrosis. Nonalcoholic fatty liver (NAFL) presents with steatosis, while nonalcoholic steatohepatitis (NASH) presents with steatosis, inflammation, and fibrosis, which can progress to the development of cirrhosis and even malignancy. [2, 3]. It was estimated that 10%–25% of patients with NASH eventually develop cirrhosis [4, 5].

Since NAFLD/NASH is associated with metabolic syndrome, its development is linked to obesity [1]. However, 8%–19% of Asians with NAFLD are not obese (body mass indices < 25 kg·m^−2^) [6]. Studies have shown that this ‘lean’ or ‘nonobese’ subtype of NAFLD is closely linked to insulin resistance, diabetes, and other metabolic complications [6, 7]. Despite previous studies, the difference between these two subtypes has not been defined. The physiological processes on how the ‘nonobese’ subtype also progresses to NASH and cirrhosis have not yet been determined [6, 7].

Dietary animal models of NASH have proven that in addition to developing insulin resistance, a high‐fat diet promotes moderate steatosis, inflammation, and fibrosis in the liver [8, 9]. It was established that a choline‐deficient, methionine‐lowered, l‐amino acid‐defined diet (CDAA) in Fischer 344 rats induced NASH with features similar to human NASH steatohepatitis, hepatic fibrosis/cirrhosis, and hepatocellular carcinoma [10, 11, 12]. The involvement of oxidative stress and signaling abnormalities has also been shown in both the CDAA model and its human counterpart [10, 11, 12]. Of note are the minimal effects on body weight and glucose metabolism by the CDAA in contrast to semi‐purified methionine‐ and choline‐deficient diets (MCDs) [10, 11, 12]. This leads to the idea that the CDAA model may serve as a good tool to assess the ‘nonobese’ NASH subtype profile.

Whereas mice were mainly resistant to CDAA [13], Matsumoto *et al*. recently developed a modified CDAA by reducing methionine content and increasing fat [choline‐deficient, methionine‐lowered, l‐amino acid‐defined high‐fat diet (CDAHFD)]. This diet induces NASH in mice [14], but its underlying mechanisms remain unknown.

This study was conducted to analyze the role of dietary methionine content in the development of NASH in mice fed with CDAHFD. The results indicate that CDAHFD with insufficient methionine induces NASH without being obese (nonobese subtype), while CDAHFD with sufficient methionine only induces NAFL despite promoting obesity. We then searched for important signal pathways in the ‘nonobese’ NASH subtype by gene profile analysis using the mice liver samples.

## Materials and methods

### Diets

The control diet of CE‐2 was composed of 58% carbohydrate, 13% fat, and 29% protein on a caloric basis, 0.21% choline, and 0.44% methionine and were obtained from CLEA Japan Inc. (Tokyo, Japan). The experimental diets of CDAHFD‐0.1 (the fat amount 45%, methionine amount 0.1%; ID A06071309) and CDAHFD‐0.6 (the fat amount 45%, methionine amount 0.6%; ID A16032501) were obtained from the Research Diet Inc. (New Brunswick, NJ, USA). The dietary components of the experimental diets are shown in Table S1. The diets were frozen before use and changed every two days to prevent oxidation.

### Animals

Five‐week‐old male C57BL/6J mice were purchased from Japan SLC (Shizuoka, Japan) and acclimated for a week before the study. The mice were kept under temperature‐controlled conditions (22°C on average) in colony cages with a 12‐h light/12‐h dark cycle and given free access to food and water during the acclimation and experimental periods. At 6 weeks of age, the mice were randomly assigned to the three groups (control diet, CDAHFD‐0.1, or CDAHFD‐0.6) for either 13 (*n* = 5–6) or 26 (*n* = 10–11) weeks. The study design is shown in Fig. S1. Body weight, food consumption, and water intake were monitored weekly. At the end of the experimental period, blood samples were collected from the tail vein of all mice. The mice were euthanized by exsanguination under light isoflurane anesthesia. All organs were carefully studied during the autopsy, and the liver and other lesion‐baring organs were excised and weighed if any needed weighing. Organ weight was normalized to body weight according to the literature [15].

### Histological analysis

Liver samples were fixed in 10% neutrally buffered formalin, embedded in paraffin, and cut into 4‐µm‐thick sections for hematoxylin–eosin (H&E) and Sirius Red staining. Histopathological diagnosis of liver lesions was conducted by a scientist unaware of the treatment of the mice and according to the INHAND criteria [16]. Using Sirius Red‐stained specimens, the areas of fibrosis were measured using a cellSens Dimension software (Olympus, Tokyo, Japan). Immunohistochemical analyses were conducted as previously described [17] with samples obtained from mice fed for 13 or 26 weeks using the following primary antibodies: rat anti‐mouse monoclonal antibody for F4/80 as a marker of macrophages (1 : 200; Abcam, Cambridge, UK), goat anti‐mouse polyclonal antibody for Clec4f as a marker of Kupffer cells (1 : 400; R&D Systems, MN, USA), rat anti‐mouse monoclonal antibody for Ly6c as a marker of infiltrating monocytes (1 : 40; R&D Systems, MN, USA), rabbit antihuman polyclonal antibody for α‐smooth muscle actin (α‐SMA) as a marker of activated hepatic stellate cells (1 : 200; Abcam, Cambridge, UK), mouse antiproliferating cell nuclear antigen (PCNA) as a marker of cellular proliferation (1 : 100; DAKO, Kyoto, Japan), and mouse antihuman monoclonal antibody for cytokeratin 8/18 (CK8/18) as a marker of putative hepatocellular preneoplastic lesions (1 : 500; Developmental Studies Hybridoma Bank, Iowa, USA). The visualization of antibody binding was performed using Histofine Simple Stain Kit (Nichirei Corp., Tokyo, Japan) for F4/80, α‐SMA, and PCNA, or a VectaStain *Elite* ABC Kit (Vector Laboratories, Burlingame, CA, USA) for CK8/18. All sections were counterstained using hematoxylin and histopathologically examined in an unaware manner. The findings were graded as normal (−), minimal (1+), moderate (2+), and severe (3+) and were assigned scores of 1, 2, 3, and 4, respectively. The numbers of CK8/18‐positive putative hepatocellular preneoplastic lesions consisting of 1, 2, 3, or more cells were counted per 10 light microscopy fields (×200).

### Plasma and hepatic chemistry

Plasma was obtained from the blood samples to measure triglyceride (TG) and total cholesterol (TC) levels, and aspartate (AST) and alanine (ALT) aminotransferase activities using either an automatic analyzer (DRI‐CHEM; Fujifilm, Tokyo, Japan) or colorimetry test kits purchased from Wako Pure Chemical Industries (Osaka, Japan). Hepatic TG and TC levels were measured as previously described [18].

### Glucose tolerance tests and insulin tolerance tests

At 8 and 25 weeks, glucose tolerance tests (GTTs) and insulin tolerance tests (ITTs) were conducted. Following overnight fasting, glucose was administered by gavage at a single dose of 1.5 g·kg^−1^ body weight for GTTs. For ITTs, insulin (Humulin R, Eli Lilly, Indianapolis, IN, USA) was injected intraperitoneally at a single dose of 0.35 U·kg^−1^ body weight. The glucose values in the blood obtained from the tail vein were measured using a glucometer system Accu‐Chek ^®^ Aviva (Roche Diagnostics, Rotkreuz, Switzerland) at 0, 15, 30, 60, and 120 min after the administration of glucose or insulin. Additionally, serum was prepared from the blood collected from the tail vein, and the insulin values were measured using commercial insulin ELISA kits (Shibayagi Co., Ltd., Gunma, Japan).

### RNA extraction and analysis

Total RNA was extracted from the liver using a Sepasol reagent (Nacalai Tesque, Kyoto, Japan) and was reverse‐transcribed using PrimeScript RT Master Kit (Takara Bio Inc., Shiga, Japan), according to the manufacturers’ instructions. Afterward, quantitative real‐time PCR (qPCR) was conducted using a SYBR Premix Ex Taq (Takara Bio Inc. Shiga, Japan) and specific primer sets with a Thermal Cycler Dice Real‐Time System Single (Takara Bio Inc. Shiga, Japan). The primer sequences for qPCR in this study are shown in Table S2. The mRNA expression levels were normalized to that of cyclophilin mRNA. Portions of the RNA samples were sequenced (RNA‐Seq) and analyzed via qPCR analyses. RNA‐Seq was conducted as described elsewhere [19]. Portions of the 100 ng of total RNA from the livers of the control, CDAHFD‐0.1, and CDAHFD‐0.6 groups (treated for 13 weeks, *n* = 3–4) were used for the library preparation. Sequencing libraries were generated using TruSeq RNA Library Preparation Kit v2 (Illumina Inc., San Diego, CA, USA). The principal component analysis (PCA), differential expression analysis, generation of heat maps with hierarchical clustering of samples, and features and functional annotation analyses using Ingenuity Pathway Analysis (IPA) software (Ingenuity Systems, Qiagen Co., Ltd, Redwood City, CA, USA) were conducted as previously described [19].

### Statistical analysis

Numerical values were expressed as means ± standard deviations (SDs). One‐way analysis of variance (ANOVA) followed by the Tukey–Kramer multiple comparisons test was used to assess differences among groups. Differences were considered significant if *P* < 0.05.

### Ethical consideration

All animal husbandry and experiments were conducted in compliance with the guiding principle of the Tokyo University of Agriculture and approved by the Animal Experiment Committee of the University. Consequently, this study complied with all related domestic and international laws, regulations, and guidelines. Particularly, animal experiments conducted in this study complied with the ARRIVE guidelines and were conducted according to the U.K. Animals (Scientific Procedures) Act 1986 and associated guidelines, EU Directive 2010/63/EU for animal experiments, and the National Institutes of Health guide for the care and use of laboratory animals (NIH Publications No. 8023, revised 1978). This study used only male mice because our previous studies have clearly demonstrated that female animals are resistant to CDAA [20].

## Results

### Physiologic, hematologic, and hepatic chemical changes

**Fig. 1:**
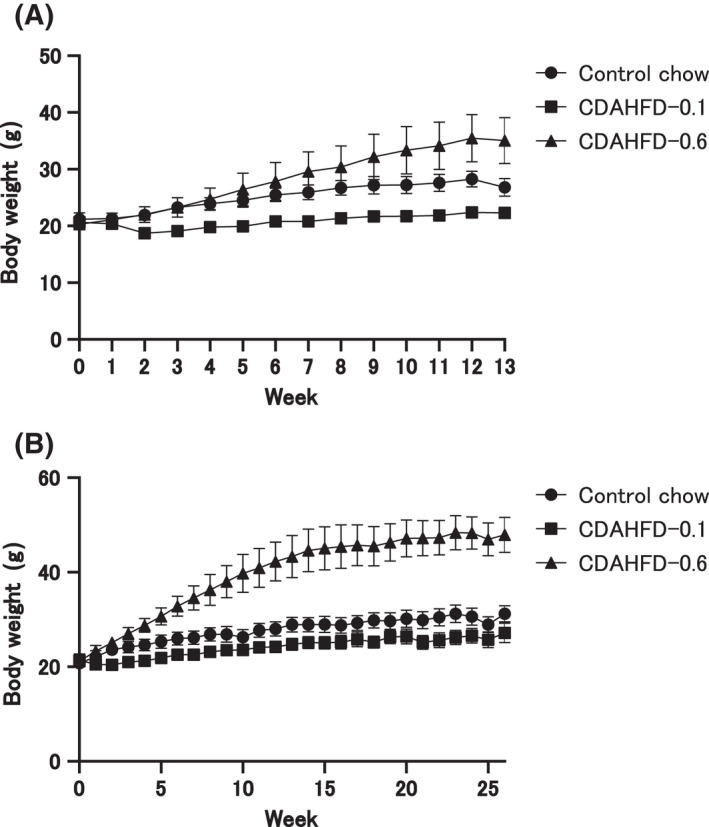
Body weight changes. Body weight changes in C57BL/6J mice fed with the control chow (*n* = 5 or *n* = 10), CDAHFD‐0.1 (*n* = 5 or *n* = 11), or CDAHFD‐0.6 (*n* = 6 or *n* = 10) for 13 (A) or 26 (B) weeks. The values are presented as the means + SDs

In the CDAHFD‐0.1 group, body weight decreased compared with the control group but recovered to baseline at week 26 (Fig. 1A,B). However, we observed a significant increase in the body weight of the CDAHFD‐0.6 mice relative to the control (Fig. 1A,B). Despite the difference in body weight, food consumption and water intake were similar in the control, CDAHFD‐0.1, and CDAHFD‐0.6 groups (data not shown). Organ weights, plasma, and hepatic chemistry at the end of week 26 are shown in Table 1. Compared to the control group, there was an increase in the relative weights of the liver and eWAT, enhanced plasma ALT, and AST activities in the CDAHFD‐0.1 group. Additionally, hepatic TG and TC levels were markedly elevated. However, CDAHFD‐0.6 was approximately equal to the relative weight of the liver, substantially increased the relative weights of the eWAT, and enhanced plasma ALT and AST activities, when compared with the values of the control group. AST activity significantly decreased compared with the CDAHFD‐0.1 group. For the plasma lipid levels, TG was similar in the control group, whereas TC markedly increased. The hepatic TG level was clearly elevated, whereas hepatic TC level increased.

**Table 1:** Organ weights and plasma and hepatic chemistries at the end of week 26. Values are means ± SDs. eWAT, epididymal white adipose tissue; TC, total cholesterol; TG, triglyceride.

	Control chow	CDAHFD‐0.1	CDAHFD‐0.6
Liver (g)	1.35 ± 0.11	1.93 ± 0.25^a^	2.25 ± 0.42^a^, ^b^
Liver/BW (w/w%)	4.33 ± 0.27	7.17 ± 1.31^a^	4.67 ± 0.64^b^
Kidney (g)	0.36 ± 0.02	0.37 ± 0.16	0.38 ± 0.02
Heart (g)	0.14 ± 0.02	0.14 ± 0.02	0.17 ± 0.01^a^, ^b^
eWAT (g)	0.74 ± 0.13	0.83 ± 0.18	1.94 ± 0.24^a^, ^b^
eWAT/BW (w/w%)	2.36 ± 0.37	3.03 ± 0.56^a^	4.09 ± 0.66^a^, ^b^
Plasma TG (mg·dL^−1^)	106.10 ± 39.39	99.22 ± 23.16	81.41 ± 25.79
Plasma TC (mg·dL^−1^)	64.50 ± 8.47	56.13 ± 20.73	165.00 ± 43.43^a^, ^b^
ALT (IU·L^−1^)	9.48 ± 6.94	45.06 ± 6.85^a^	48.57 ± 29.03^a^
AST (IU·L^−1^)	26.72 ± 10.73	112.01 ± 20.10^a^	71.38 ± 29.63^a^, ^b^
Liver TG (mg·g^−1^)	19.45 ± 5.48	85.17 ± 7.45^a^	83.84 ± 25.08^a^
Liver TC (mg·g^−1^)	3.34 ± 0.23	9.04 ± 2.83^a^	5.34 ± 3.34

^a^ Significantly different from the control value.

^b^ Significantly different from the CDAHFD‐0.1 value.

### Morphological changes and their relating mRNA expression profiles

**Fig. 2:**
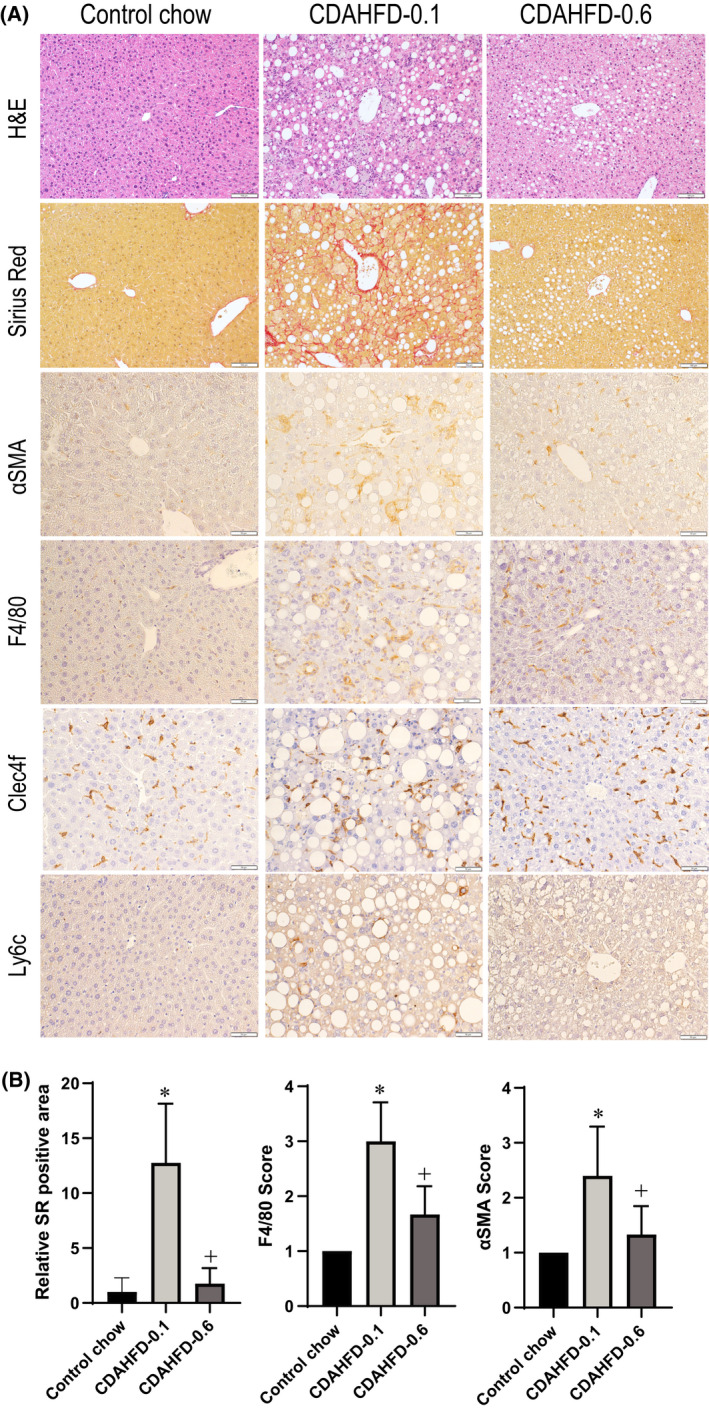
Representative histopathology and immunohistochemistry of the liver at the end of week 13. Representative features for H&E, Sirius Red, α‐SMA, F4/80, Clec4f, and Ly6c staining (A), and Sirius Red‐positive area and scores for F4/80 and α‐SMA (B). The lengths of the scale bars are 100 µm (H&E and Sirius Red) and 50 µm (α‐SMA, F4/80, Clec4f, and Ly6c). The values are presented as the means + SDs on the control (*n* = 5), CDAHFD‐0.1 (*n* = 5), and CDAHFD‐0.6 (*n* = 6) groups. Difference between the means was statistically determined significant when *P* < 0.05, using one‐way ANOVA followed by the Tukey–Kramer multiple comparisons test. *Significantly different from the control group value. ^+^Significantly different from the CDAHFD‐0.1 group value.

The representative microscopic features of nonproliferative liver lesions and their grading scores are shown in Fig. 2A,B. At the end of week 13, macrovesicular steatosis characterized by hepatocytes with a single large cytoplasmic vacuoles was observed in almost all hepatocytes in the CDAHFD‐0.1 group. Inflammatory clusters consisting of markedly accumulated Kupffer cells and hypertrophied macrophages were visualized using F4/80 immunohistochemistry. Immunohistochemical staining of liver sections for Clec4f, a specific Kupffer cell marker, showed positive cells in all groups. While the number of positive cells was similar among the three groups, morphological differences were observed. Namely, Clec4f staining revealed that positive cells appeared to form the hepatic crown‐like structure derived from resident macrophages and reported to be involved in fibrosis in NASH [21, 22], in the CDAHFD‐0.1 group, but not in the control or CDAHFD‐0.6 group. Immunohistochemical staining of liver sections for Ly6c, a specific infiltrating monocyte marker, demonstrated partially stained hypertrophic macrophages and foamy macrophages, only in the CDAHFD‐0.1 group, and not in the livers of the control or CDAHFD‐0.6 group. The number of activated stellate cells and Sirius Red‐positive areas was elevated. However, only macrovesicular and microvesicular steatosis were observed in the CDAHFD‐0.6 group. The magnitudes of fibrosis and the immunohistochemical α‐SMA, F4/80, and Ly6c reactivities were greater in the CDAHFD‐0.1 group than in the control and the CDAHFD‐0.6 groups.

**Fig. 3:**
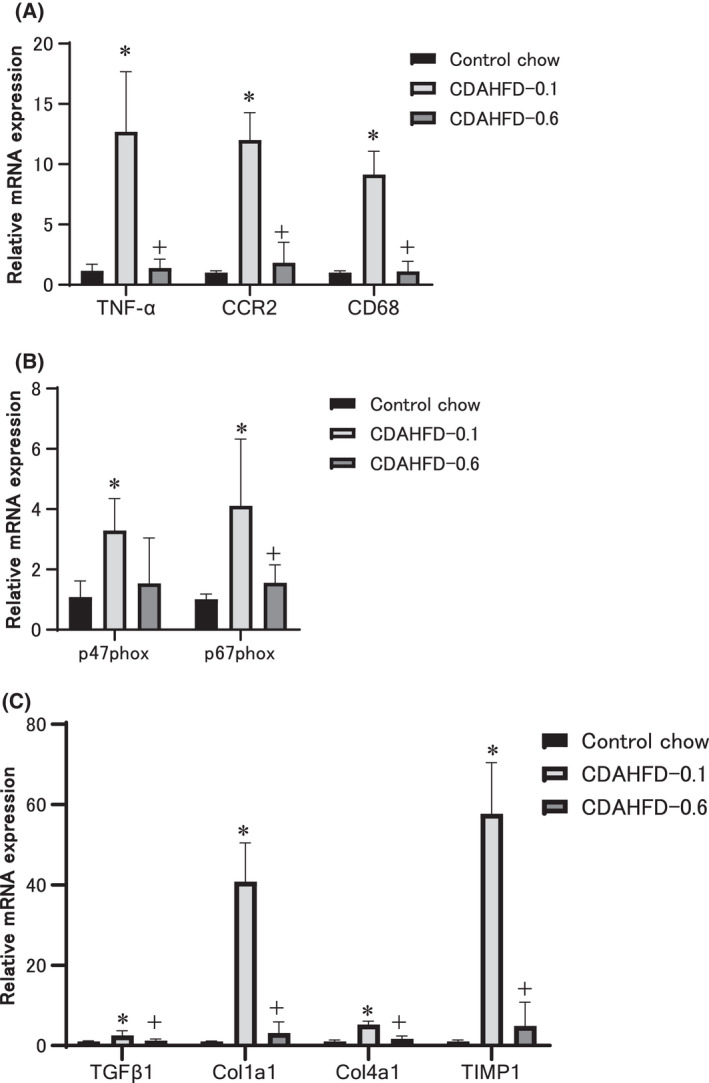
mRNA expression levels of gene‐related morphological changes in the liver at the end of week 13. qPCR of genes involved in inflammation (A), oxidative stress (B), and fibrosis (C) in the livers of mice fed the control chow (*n* = 5), CDAHFD‐0.1 (*n* = 5) or CDAHFD‐0.6 (*n* = 6), for 13 weeks. The values are presented as the means + SDs. Difference between the means was statistically determined significant when *P* < 0.05, using one‐way ANOVA followed by the Tukey–Kramer multiple comparisons test. *Significantly different from the control group value. ^+^Significantly different from the CDAHFD‐0.1 group value.

The mRNA expressions reflected the morphological features. The expression of inflammation‐related markers, such as tumor necrosis factor‐α (TNF‐α), C‐C motif chemokine receptor 2 (CCR‐2), and the cluster of differentiation 68 (CD68), was strongly upregulated by CDAHFD‐0.1 but not CDAHFD‐0.6 (Fig. 3A). The expression of genes associated with reactive oxygen species production, p47phox, and p67phox was also dramatically upregulated by CDAHFD‐0.1 but not CDAHFD‐0.6 (Fig. 3B). Moreover, gene expression associated with fibrosis, such as transforming growth factor β1 (TGFβ1), collagen type 1 α1 (Col1a1), collagen type 4 α1 (Col4a1), and tissue inhibitor of metalloproteinase 1 (TIMP1), was markedly increased by CDAHFD‐0.1 but not CDAHD‐0.6 (Fig. 3C). CDAHFD‐0.6 did not alter the mRNA expression of any assessed gene.

At the end of week 26 in the CDAHFD‐0.1 group, fibrosis progressed (Fig. S2A), while steatosis and inflammation remained virtually unchanged. In contrast, the CDAHFD‐0.6 group still did not develop fibrosis (Fig. S2A). The mRNA expression showed that the upregulation of inflammatory and fibrosis markers progressed further at the end of the week 26 in the CDAHFD‐0.1 group. However, the CDAHFD‐0.6 group also showed progression but with a lesser magnitude than the CDAHFD‐0.1 group (Fig. S2B‐D).

### Insulin reactivity

**Fig. 4:**
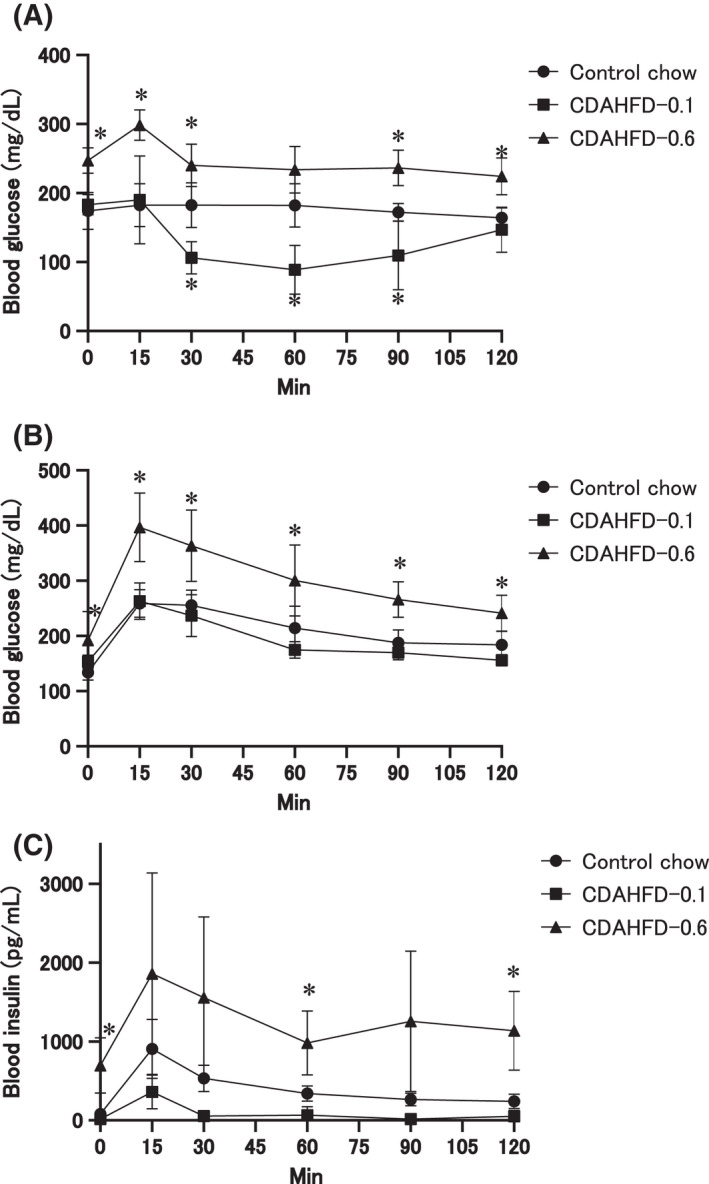
Evaluation of glucose tolerance and insulin sensitivity. Blood glucose levels in (A) ITT and (B) OGTT tests, and blood insulin levels in OGTT tests (C) at the end of week 26 on the control (*n* = 5), CDAHFD‐0.1 (*n* = 5), and CDAHFD‐0.6 (*n* = 6) groups. The values are presented as the means + SDs. Difference between the means was statistically determined significant when *P* < 0.05, using one‐way ANOVA followed by the Tukey–Kramer multiple comparisons test. *Significantly different from the control group value.

OGTT and ITT at the end of week 26 showed that the mice fed with CDAHFD‐0.1 were remarkably sensitive to insulin (Fig. 4A–C). In fact, the ITT test, which would have administered 0.5 U·kg^−1^ of insulin, had to be discontinued because of a marked decrease in blood glucose levels (data not shown). As a result, the ITT test was conducted by administering a lower dose of 0.35 U·kg^−1^ in this second trial, which also showed decreased blood glucose in the CDAHFD‐0.1, as compared to the control group. In contrast, the glucose level was consistently elevated in the CDAHFD‐0.6 group compared with the control group (Fig. 4A). In the OGTT test, the blood glucose profiles in the CDAHFD‐0.1 group were similar to those in the control group (Fig. 4B), while the insulin levels tend to be lower in the former than those in the latter (Fig. 4C). However, blood glucose and insulin levels were significantly elevated in the CDAHFD‐0.6 group (Fig. 4B,C). When the ITT test was conducted at the end of week 8, insulin sensitivity was already enhanced in the CDAHFD‐0.1 group, while insulin resistance was induced in the CDAHFD‐0.6 group (Fig. S3). From the results, it was determined that CDAHFD‐0.1 induced insulin sensitivity, while CDAHFD‐0.6 induced insulin resistance, and the different changes were induced regarding sensitivity to insulin by CDAHFD‐0.1 and CDAHFD‐0.6 as early as 8 weeks and continued thereafter.

### Development of proliferative hepatic lesions

**Fig. 5:**
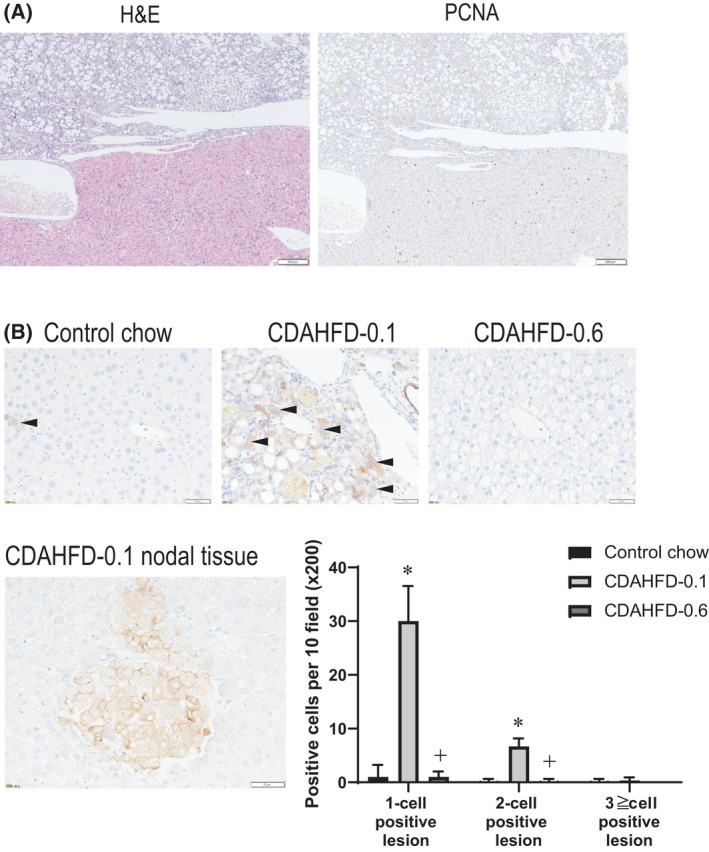
Representative proliferative hepatic lesions of the liver at the end of week 26. Microscopic features of a hepatic proliferative lesion (in the lower part) and its adjacent liver tissue (in the upper part) of H&E and PCNA staining of mice fed CDAHFD‐0.1 (A). The immunohistochemistry of CK8/18 (arrows indicating positive cells), numbers of CK8/18‐positive, and putative hepatocellular preneoplastic lesions (B). The lengths of the scale bars are 200 µm (A) and 50 µm (B). The values are presented as the means + SDs on the control (*n* = 10), CDAHFD‐0.1 (*n* = 11) and CDAHFD‐0.6 (*n* = 10) groups. Difference between the means was statistically determined significant when *P* < 0.05, using one‐way ANOVA followed by the Tukey–Kramer multiple comparisons test. *Significantly different from the control group value. ^+^Significantly different from the CDAHFD‐0.1 group value.

Table 2 shows the incidence of hepatocellular proliferating lesions at the end of week 26. The foci of cellular alteration, proliferative, and preneoplastic [16] were observed in 9/11 mice (82%) and 0/10 mice (0%) in the CDAHFD‐0.1 and CDAHFD‐0.6 groups, respectively. Regenerative hepatocellular hyperplasia, proliferative, but not necessarily preneoplastic [16], were observed in 2/11 mice (18%) and 0/10 mice (0%) in the CDAHFD‐0.1 and CDAHFD‐0.6 groups, respectively. Representative histopathology of regenerative hepatocellular hyperplasia in the CDAHFD‐0.1 group is shown in Fig. 5A. PCNA‐positive cells were frequently found in this proliferative lesion (Fig. 5A). Apparent proliferative lesions were not observed in the CDAHFD‐0.1 group at the end of week 13, but PCNA‐positive cells were clearly increased compared with the control chow group, suggesting that the hepatocellular proliferation is induced within 13 weeks. In the CDAHFF‐0.6 group, there was also an increase in PCNA‐positive cells, compared to the control group, but with a lesser magnitude (Fig. S4).

**Table 2:** Incidences of hepatocellular proliferative lesions at the end of week 26.

Lesion	Control chow	CDAHFD‐0.1	CDAHFD‐0.6
Foci of cellular alteration	0 (0)	9 (82)	0 (0)
Hyperplasia, hepatocellular, regenerative	0 (0)	2 (18)	0 (0)

The number of hepatocytes immunohistochemically positive for CK8/18, a marker for mouse preneoplastic hepatocellular lesions [23], was significantly higher in the CDAHFD‐0.1 group than in the control and CDAHFD‐0.6 groups at the end of week 26 (Fig. 5B) and also at that of week 13 (Fig. S4). CK8/18‐positive foci were observed in the CDAHFD‐0.1 group, and they corresponded to histologically detected foci of cellular alteration (Fig. 5B). In all specimens, CK8/18 was positive in the bile duct epithelial cells, which indicates that the staining was successful.

### Gene expression profiles at the end of week 13

**Fig. 6:**
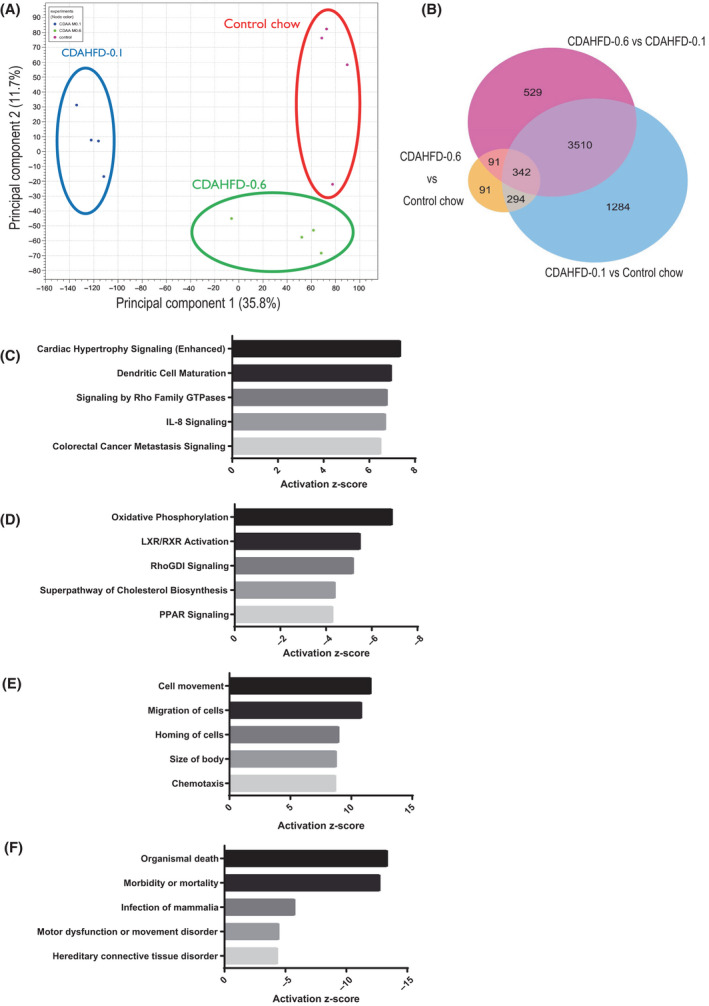
Gene expression profiles at the end of week 13. Two‐dimensional plot of the principal component analysis for RNA‐Seq (A) and the Venn diagram of the comparison of DEGs based on RNA‐Seq data (B). The genes were selected using the expression analysis for comparisons between the control, CDAHFD‐0.1, and CDAHFD‐0.6 groups, with an FDR *P* value < 0.05 and/or FC > ±1.5. Activated disease or functional annotation (|z‐score| ≥ 2) for DEGs in Ingenuity Pathway Analysis. Top 5 of upregulated (C) or downregulated (D) in the canonical pathway. Top 5 of upregulated (E) or downregulated (F) in the disease and biofunction.

RNA‐Seq was conducted using liver samples obtained at the end of week 13. PCA was conducted to identify outlier samples for quality control and determine the primary causes of variation in the dataset (Fig. 6A). The CDAHFD‐0.1 and CDAHFD‐0.6 groups were separated by the first principal component (35.8%, horizontal axis). The second principal component (11.7%, vertical axis) separated the control and CDAHFD‐0.6 groups. The analyses of various differentially expressed genes (DEGs) were conducted between the control and either of the CDAHFD‐0.1 or CDAHFD‐0.6 group under the conditions of a false discovery rate (FDR) *P* value < 0.05 and fold change (FC) > ±1.5. The Venn diagram of these genes is shown in Fig. 6B. The DEGs for the control versus CDAHFD‐0.1, the control versus CDAHFD‐0.6, and the CDAHFD‐0.6 versus CDAHFD‐0.1 were 5430, 818, and 4472, respectively. To identify specifically altered pathways and disease states in severe NASH without obesity and NAFL with obesity, the functional analysis of the DEGs using IPA was conducted from 4472 genes of which expressions were differentially altered between the CDAHFD‐0.6 and CDAHFD‐0.1 groups. In the IPA analysis of the canonical pathway and the disease and biofunction, the top 5 upregulated or downregulated pathways with z‐score > ±2 were shown in Fig. 6C–F. In the canonical pathway of the CDAHFD‐0.1 group, genes related to cardiac hypertrophy, Rho family GTPases, and immune system processes, such as dendritic cell maturation and IL‐8 signaling, were upregulated (Fig. 6C), while those related to oxidative phosphorylation, RhoGDI, and lipid metabolism, such as LXRs/RXRs, peroxisome proliferator‐activated receptor (PPAR) activation, and cholesterol synthesis, were downregulated (Fig. 6D). In the disease and biofunction of the CDAHFD‐0.1 group, genes related to cellular movement, such as migration and homing and chemotaxis, were upregulated (Fig. 6E), while those related to cell death and cell motility disorders were downregulated (Fig. 6F). Details of these selected signaling pathways by IPA analysis and all the DEGs are listed in Tables S3 and S4, respectively.

## Discussion

This study assessed the effects of dietary methionine content on inducing NASH in mice fed with CDAHFD and to investigate important signaling pathways in the ‘nonobese’ subtype of NASH. The CDAHFD‐0.1 group with insufficient methionine content increased insulin sensitivity and induced severe ‘nonobese’ NASH characterized by inflammation, fibrosis, and the development of proliferative lesions in the liver. In contrast, the CDAHFD‐0.6 group with sufficient methionine content only developed NAFL with little to no inflammation or fibrosis despite inducing obesity and insulin resistance. This indicated that dietary methionine content may play a critical role in the manifestation of obesity and NASH in mice. Insulin resistance and obesity may not necessarily be essential as previously thought for developing NASH. Additionally, the livers of the CDAHFD‐0.1 group showed upregulation of Rho GTPases signaling, reduction in oxidative phosphorylation, and a defect in nuclear receptor signaling. It is suggested that these changes may be involved in the development of ‘nonobese’ NASH subtype.

Phosphatidylcholine (PC), derived from choline, plays a vital role in the hepatic secretion of very low‐density lipoprotein (VLDL), which is rich in hydrophobic triglycerides [24]. Impaired synthesis of PC due to choline deficiency will cause an accumulation of lipids in the liver. Another source of PC is methionine [25]. Histopathological examination revealed that CDAHFD‐0.1 caused severe NASH, while CDAHFD‐0.6 induced only mild NAFL. The biochemical tests showed that both CDAHFD‐0.1 and CDAHFD‐0.6 markedly increased liver TG, whereas liver cholesterol levels were elevated only in CDAHFD‐0.1. Some studies have linked altered hepatic cholesterol homeostasis and cholesterol accumulation to the pathogenesis of NASH [26]. Therefore, it is suggested that the accumulation of cholesterol in the liver in the CDAHFA‐0.1 group is partly involved in the development of NASH and that the supplementation of methionine in the CDAHFD‐0.6 group prevented hepatic cholesterol accumulation, preventing the progression of NAFL to NASH. As an essential amino acid, methionine serves as a methyl donor, acts as a precursor of antioxidant enzymes and polyamines, and is widely involved in liver function [27]. As exhibited by the CDAHFD‐0.1 group, the deficiency of methionine can disturb liver function and promote an oxidative microenvironment that may progress to NASH. There was marked weight gain in CDAHFD‐0.6 compared with CDAHFD‐0.1, which may be interpreted as an increase in the nutritional efficiency of ingested food due to sufficient amounts of methionine. Previous studies have shown that dietary restriction of methionine will inhibit weight gain [28], and methionine and choline play essential roles in weight management in mice [29].

In this study, CDAHFD‐0.1 showed morphological changes in Kupffer cells and increased infiltrating macrophages. A previous study showed that infiltrated macrophages expressed CD68, CCR‐2, and Ly6c and that CCR‐2^−/−^ mice had less inflammatory cell infiltration and hepatic fibrosis, suggesting that hepatic recruitment of macrophages promotes NASH through CCR‐2 [30]. Additionally, resident macrophages of CD11c+ constitute crown‐like structures (hCLS), induced in the mouse model of human NASH, and this hCLS is involved in the development of hepatic inflammation and fibrosis, thereby suggesting its pathophysiologic role in disease progression from simple steatosis to NASH [21, 22]. Because the CDAHFD‐0.1 group showed changes in both resident macrophages and infiltrated bone marrow‐derived macrophages, it was indicated that CDAHFD‐0.1 causes dynamic changes in macrophages in the liver. However, CDAHFD‐0.6 showed little change compared with the control at the end of week 13 and only a slight change at that of week 26. Therefore, methionine levels in CDAFHD have a strong effect on the macrophage profile in the liver.

In this study, the CDAHFD‐0.1 showed enhanced peripheral insulin sensitivity features, as indicated by lowered ITT and OGTT glucose excursion curves. A disadvantage in the MCD diet is the induction of hypophagia and hypercatabolism, resulting in significant body weight loss with a proportional loss of liver mass [31]. This suggests that characteristics of the liver‐centric MCD effects are well‐known to induce a progressive hypercatabolic state, which promotes a wasting‐like response characterized by reduced plasma insulin levels with enhanced peripheral insulin sensitivity and mobilization of hepatic glycemic stores to maintain normoglycemia in the context of undernutrition. In this study, CDAHFD‐0.1 exhibited transient weight loss, but it recovered, and there was no decrease in eWAT weight. Therefore, it is possible that the increased insulin sensitivity was caused by a response to nutritional deficiency similar to MCD. Still, it is also possible that it was caused by NASH progression, such as changes in liver signaling.

RNA‐Seq revealed that the gene expression profile of the CDAHFD‐0.1 group was remarkably altered from those of the control and CDAHFD‐0.6 groups. Sequencing also revealed that there were minimal changes between the control and CDAHFD‐0.6 groups. As a result of canonical pathway analysis using genes with specific changes in the CDAHFD‐0.1 group, genes related to the Rho family GTPases were upregulated. GTPases of the Rho family are molecular switches that convert and amplify external signals into cellular effects, and the protein family members are divided into Rac, cdc42, Rho, and other subfamilies [32, 33]. Additionally, genes related to Rho GDI were downregulated in the CDAHFD‐0.1 group. Rho GDI, a negative regulator of Rho GTPases, extracts Rho GTPases from cell membranes and prevents their activation [34]. With this in mind, Rho GTPase signaling should be activated in the CDAHFD‐0.1 group. Rho GTPases have been implicated in diverse cellular processes that influence cell proliferation, differentiation, motility, adhesion, survival, and secretion [33, 35, 36, 37]. The upregulation of the Rho family GTPases pathway in the CDAHFD‐0.1 group influences the upregulation of cardiac hypertrophy signaling, activation of signaling relating to cell movement, migration, homing, body size, and chemotaxis, and suppression of cell death signaling. Dendritic cell maturation and IL‐8 signaling were activated in the CDAHFD‐0.1 group, both of which have also been reported to activate Rho GTPases signaling. [38, 39]. Rho GTPases play key roles in processes, such as cell cycle progression, cell survival, and gene expression, and their deregulation is thought to lead to hepatocellular tumorigenesis [40]. Therefore, studies suggest that enhanced Rho GTPases signaling might serve as a driving force for developing hepatocellular proliferative lesions in the CDAHFD‐0.1 group. While the relationship between Rho GTPases and NASH has yet to be determined, gene profiling analysis using human NAFLD samples has revealed that Rho GTPase signaling is associated with the progression of fibrosis [41]. Rho GTPase also mediates key processes, such as glucose uptake into the skeletal muscle, adipose tissues, and muscle mass regulation [42]. In this study, the activation of the Rho GTPases signaling in the liver may be involved in the enhancement of insulin sensitivity in CDAHFD‐0.1. As stated, insulin resistance is a known risk factor for NASH in humans, but its relationship to the ‘nonobese’ NASH subtype remains undefined. Further studies are needed to clarify the involvement of insulin sensitivity with NAFLD/NASH. Taken together, the activation of Rho GTPases signaling may play an important role in the mechanisms underlying the development and progression of NASH in mice fed with CDAHFD‐0.1, and thus, Rho GTPases attract interest as preventive/therapeutic targets to control ‘nonobese’ NASH.

In contrast, CDAHFD‐0.1 indicated a decrease in mitochondrial function and lipid metabolism‐related factors. In human NASH, mitochondrial dysfunction in the liver leads to reduced adenosine triphosphate (ATP) [43] and increases oxidative stress, leading to dyslipidemia, overproduction of cytokines, cell death, inflammation, and fibrosis. For this reason, mitochondrial dysfunction is considered partly responsible for the pathogenesis of NAFLD/NASH [44]. This agrees with the present data regarding the downregulation of the oxidative phosphorylation signaling pathway in the CDAHFD‐0.1 group.

This study also identified the downregulation of the signaling pathway involved in PPAR and liver X receptors (LXRs)/RXR activation in the CDAHFD‐0.1 group. The ligand‐activated transcription factors belonging to the PPAR family are involved in energy homeostasis and, therefore, are expected to be attractive targets for obesity, obesity‐induced inflammation, and metabolic syndrome. PPARs also have anti‐inflammatory properties via the interference of the proinflammatory transcription factors and gene expressions [45, 46, 47]. In the livers of NASH patients, the expression of PPAR‐α is decreased, which is negatively correlated with the presence and severity of the disease [48]. It is also reported that in rodents, PPAR‐α and PPAR‐β/δ agonists exert protective effects against steatosis, inflammation, and fibrosis in the liver [49, 50]. The LXRs are members of the nuclear receptor superfamily and bind to the DNA of genes as obligate heterodimers complexed with retinoid X receptors (RXRs) [51]. LXRs/RXRs act as oxysterol receptors to regulate cholesterol efflux and catabolism and stimulate hepatic lipogenesis [52, 53, 54]. In this study, the canonical pathway analysis revealed that CDAHFD‐0.1 downregulated the signaling pathway involved in cholesterol synthesis, which is likely a result of negative feedback due to increased liver cholesterol content. It was also suggested that the accumulation of hepatic cholesterol in the CDAHFD‐0.1 group may be caused not only by the disturbance of the secretion of VLDL from the liver but also by preventing cholesterol efflux via the LXRs/RXR pathway. LXRs normally suppress inflammation; however, excessive activation of LXRs results in an accumulation of lipids [55, 56]. While an inverse agonist of LXRs was reported to be effective in inhibiting fatty liver and inflammation [57, 58], the significance and precise roles of LXRs in NASH remain unclear.

PPARs are involved in mitochondrial metabolism, such as fatty acid oxidation, circadian control, *de novo* lipogenesis, and gluconeogenesis [59]. Studies have reported that Rho GTPases and PPARs exhibit an inverse relationship [60, 61], and interactions among nuclear receptors, such as LXRs and PPARs, are related to metabolism exist [62]. Collectively, mitochondrial dysfunction, Rho GTPases, and defects in nuclear receptor signaling are associated with the NASH phenotype, as demonstrated in the liver of mice fed with CDAHFD‐0.1.

While CDAHFD‐0.1 mice exhibit histological hallmarks of NASH and hypercholesterolemia, the model lacks essential clinical and metabolic features in normal‐weight/BMI NASH patients, often characterized using elevated fasting blood glucose, higher rate of diabetes, and hypertriglyceridemia. However, CDAHFD‐0.1 mice are advantageous for addressing molecular features associated with NASH pathogenesis and evaluating intrahepatic effects of treatment interventions preventing the progression of NASH and development of hepatocellular carcinoma. Some of the results have already been reported in NASH in humans, as described above. Therefore, we believe that the currently revealed signal changes are helpful in elucidating mechanisms underlying human nonobese NASH with inflammation, fibrosis, and neoplastic lesions.

In conclusion, dietary methionine content plays a vital role in developing obesity and NASH in mice fed with CDAHFD. Additionally, the severity of the disease in CDAHFD with insufficient methionine may partially be due to the upregulated Rho family GTPases pathway, suggesting the relevance of mitochondrial and nuclear receptor signal dysfunction. The signaling factors/pathways detected in this study may provide fundamental information serving as novel molecular targets to control NASH, especially in its ‘nonobese’ subtype.

## Conflict of interest

The authors declare no conflict of interest.

## Author contributions

NK designed the study. NK, AA, KU, SN, and MY acquired the data. NK, SO, AW, RS, and KM analyzed and interpreted the data. NK and DN wrote the manuscript. All authors agreed to submit the manuscript.

## Supporting information


**Fig S1.** Schematic overview of the experimental design. After 1 week of acclimation, mice were divided into 3 groups and fed with the control chow, CDAHFD‐0.1, and CDAHFD‐0.6 for 13 or 26 weeks. The liver samples from the 13‐week study were used for the RNA‐Seq. analysis.

**Fig S2.** Representative histopathology and mRNA expression levels in the liver at the end of week 26. Representative features for Sirius Red (A), and qPCR of genes involved in inflammation (B), oxidative stress (C) and fibrosis (D) in the livers of mice fed the control chow (*n* = 10), CDAHFD‐0.1 (*n* = 11) or CDAHFD‐0.6 (*n* = 10) for 26 weeks. The lengths of the scale bars are 100 µm. The values are presented as the means + SDs. Difference between the means was statistically determined significant when *P* < 0.05, using one‐way ANOVA followed by the Tukey–Kramer multiple comparisons test. *Significantly different from the control group value. ^+^Significantly different from the CDAHFD‐0.1 group value.

**Fig S3.** Evaluation of insulin sensitivity. Blood glucose levels in ITT test at the end of week 8 on the control (*n* = 4), CDAHFD‐0.1 (*n* = 4) and CDAHFD‐0.6 (*n* = 4) groups. The values are presented as the means + SDs. Difference between the means was statistically determined significant when *P* < 0.05, using one‐way ANOVA followed by the Tukey–Kramer multiple comparisons test. *Significantly different from the control group value.

**Fig S4.** Representative proliferative changes of the liver at the end of week 13. The immunohistochemistry of PCNA (A) and CK8/18 (B). Arrows indicating positive cells. The values are presented as the means + SDs on the control (*n* = 5), CDAHFD‐0.1 (*n* = 5) and CDAHFD‐0.6 (*n* = 6) groups. Difference between the means was statistically determined significant when *P* < 0.05, using one‐way ANOVA followed by the Tukey–Kramer multiple comparisons test. *Significantly different from the control group value. ^+^Significantly different from the CDAHFD‐0.1 group value.

**Table S1.** Compositions of experimental diets used in this study.
Table S2. Sequence information of primers for the qPCR analyses.

**Table S3.** CDAHFD‐0.6 versus CDAHFD‐0.1: Canonical pathway analysis of differentially expressed genes by IPA; and CDAHFD‐0.6 versus CDAHFD‐0.1: Canonical pathway analysis of differentially expressed genes by IPA.

**Table S4.** Gene expression in livers of the control, CDAHFD‐0.1 and CDAHFD‐0.6 groups at the end of week 13. The following expression data are shown per sample. (1) Total counts, (2) Reads Per Kilobase of transcript per Million mapped reads (RPKM), (3) Transcripts Per Million (TPM) and (4) Counts Per Million (CPM).

## Data Availability

The original data are available upon reasonable request.
