# Supplementary material for: Nonobese mice with nonalcoholic steatohepatitis fed on a choline‐deficient, l‐amino acid‐defined, high‐fat diet exhibit alterations in signaling pathways
Source: FEBS Open Bio. 2021 Sep 21;11(11):2950–65. doi: 10.1002/2211-5463.13272 (PMC8564345; doi:10.1002/2211-5463.13272)
Supplement: Supplementary file 1 — Fig S1. Schematic overview of the experimental design. After 1 week of acclimation, mice were divided into 3 groups and fed with the control chow, CDAHFD‐0.1, and CDAHFD‐0.6 for 13 or 26 weeks. The liver samples from the 13‐week study were used for the RNA‐Seq. analysis. [file FEB4-11-2950-s003.pdf]

# Supplemental Figure S1

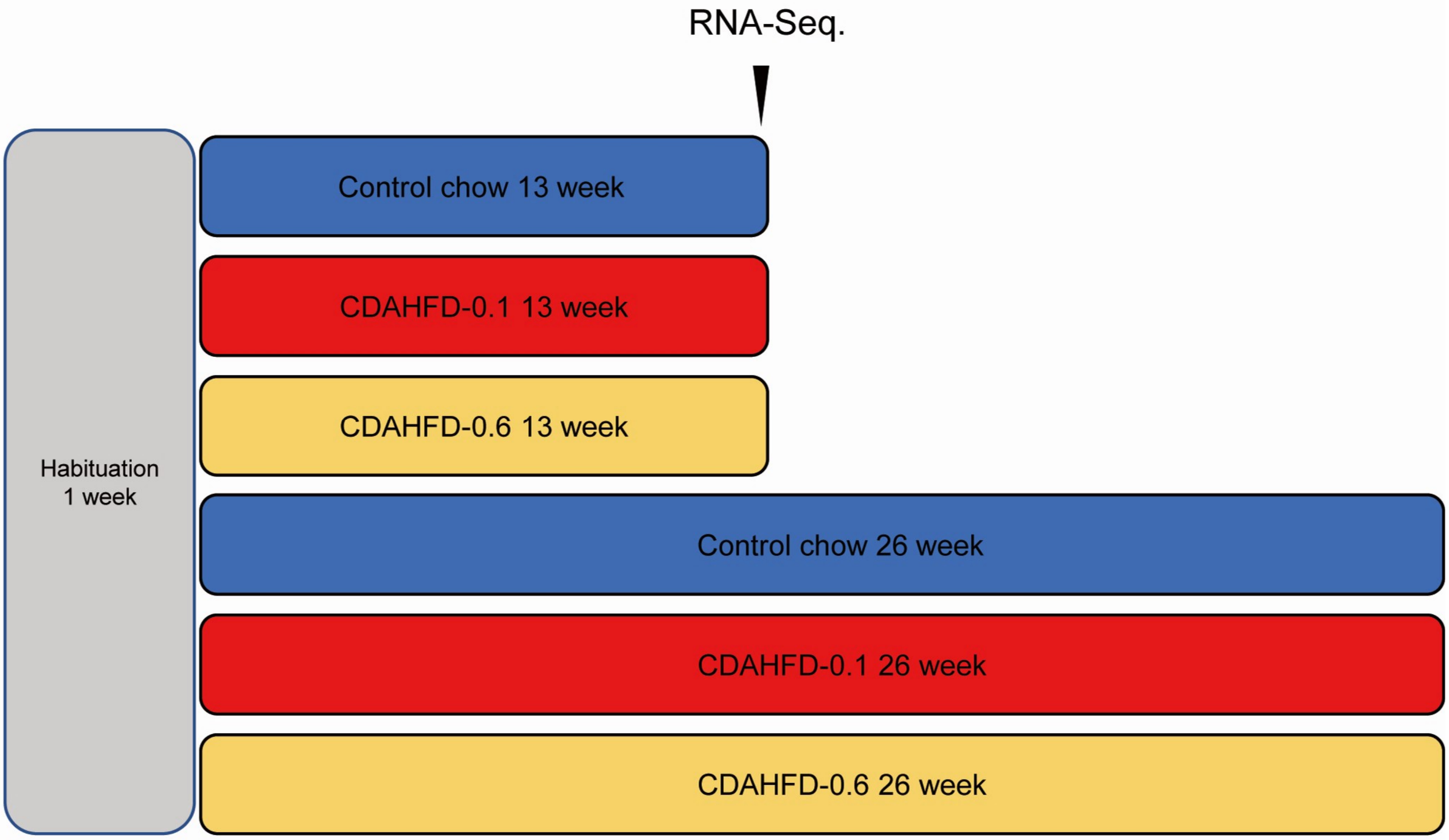


Supplemental Figure S1: Schematic overview of the experimental design. After 1 week of acclimation, mice were divided into 3 groups and fed with the control chow, CDAHFD-0.1, and CDAHFD-0.6 for 13 or 26 weeks. The liver samples from the 13-week study were used for the RNA-Seq. analysis.
