# Supplementary material for: Nonobese mice with nonalcoholic steatohepatitis fed on a choline‐deficient, l‐amino acid‐defined, high‐fat diet exhibit alterations in signaling pathways
Source: FEBS Open Bio. 2021 Sep 21;11(11):2950–65. doi: 10.1002/2211-5463.13272 (PMC8564345; doi:10.1002/2211-5463.13272)
Supplement: Supplementary file 2 — Fig S2. Representative histopathology and mRNA expression levels in the liver at the end of week 26. Representative features for Sirius Red (A), and qPCR of genes involved in inflammation (B), oxidative stress (C) and fibrosis (D) in the livers of mice fed the control chow (n = 10), CDAHFD‐0.1 (n = 11) or CDAHFD‐0.6 (n = 10) for 26 weeks. The lengths of the scale bars are 100 µm. The values are presented as the means + SDs. Difference between the means was statistically determined significant when P < 0.05, using one‐way ANOVA followed by the Tukey–Kramer multiple comparisons test. *Significantly different from the control group value. +Significantly different from the CDAHFD‐0.1 group value. [file FEB4-11-2950-s001.pdf]

Supplemental Figure S2

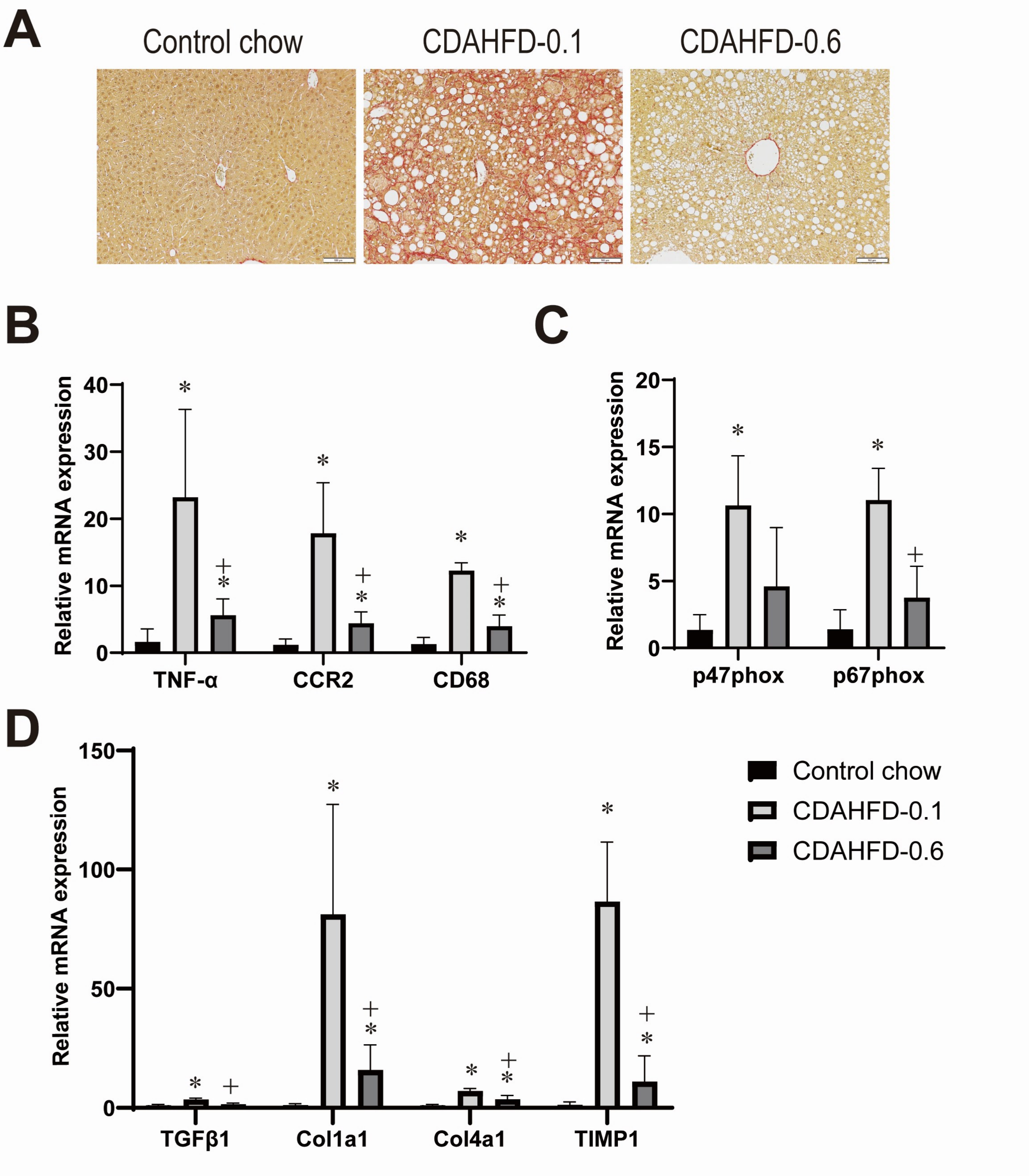

Supplemental Figure S2: Representative histopathology and mRNA expression levels in the liver at the end of week 26. Representative features for Sirius Red (A), and qPCR of genes involved in inflammation (B), oxidative stress (C) and fibrosis (D) in the livers of mice fed the control chow, CDAHFD-0.1 or CDAHFD-0.6 for 26 weeks.

*Significantly different from the control group value.

+Significantly different from the CDAHFD-0.1 group value.
