# Supplementary material for: Nonobese mice with nonalcoholic steatohepatitis fed on a choline‐deficient, l‐amino acid‐defined, high‐fat diet exhibit alterations in signaling pathways
Source: FEBS Open Bio. 2021 Sep 21;11(11):2950–65. doi: 10.1002/2211-5463.13272 (PMC8564345; doi:10.1002/2211-5463.13272)
Supplement: Supplementary file 3 — Fig S3. Evaluation of insulin sensitivity. Blood glucose levels in ITT test at the end of week 8 on the control (n = 4), CDAHFD‐0.1 (n = 4) and CDAHFD‐0.6 (n = 4) groups. The values are presented as the means + SDs. Difference between the means was statistically determined significant when P < 0.05, using one‐way ANOVA followed by the Tukey–Kramer multiple comparisons test. *Significantly different from the control group value. [file FEB4-11-2950-s006.pdf]

Supplemental Figure S3

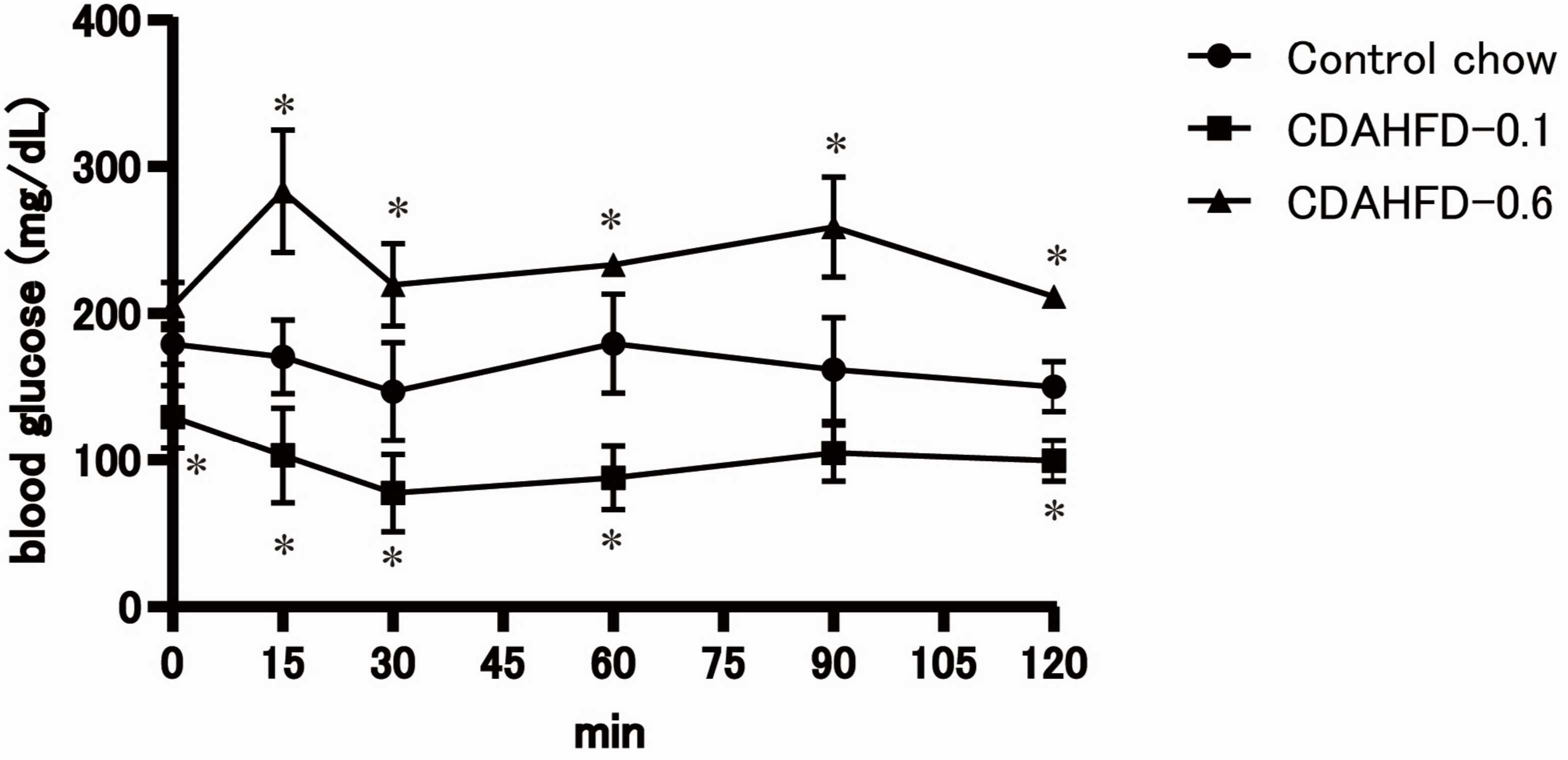

Supplemental Figure S3: Evaluation of insulin sensitivity.

Blood glucose levels in ITT test at the end of week 8

on the control (n = 4), CDAHFD-0.1 (n = 4) and CDAHFD-0.6 (n = 4) groups.

*Significantly different from the control group value.
