# Supplementary material for: Nonobese mice with nonalcoholic steatohepatitis fed on a choline‐deficient, l‐amino acid‐defined, high‐fat diet exhibit alterations in signaling pathways
Source: FEBS Open Bio. 2021 Sep 21;11(11):2950–65. doi: 10.1002/2211-5463.13272 (PMC8564345; doi:10.1002/2211-5463.13272)
Supplement: Supplementary file 4 — Fig S4. Representative proliferative changes of the liver at the end of week 13. The immunohistochemistry of PCNA (A) and CK8/18 (B). Arrows indicating positive cells. The values are presented as the means + SDs on the control (n = 5), CDAHFD‐0.1 (n = 5) and CDAHFD‐0.6 (n = 6) groups. Difference between the means was statistically determined significant when P < 0.05, using one‐way ANOVA followed by the Tukey–Kramer multiple comparisons test. *Significantly different from the control group value. +Significantly different from the CDAHFD‐0.1 group value. [file FEB4-11-2950-s004.pdf]

Supplemental Figure S4

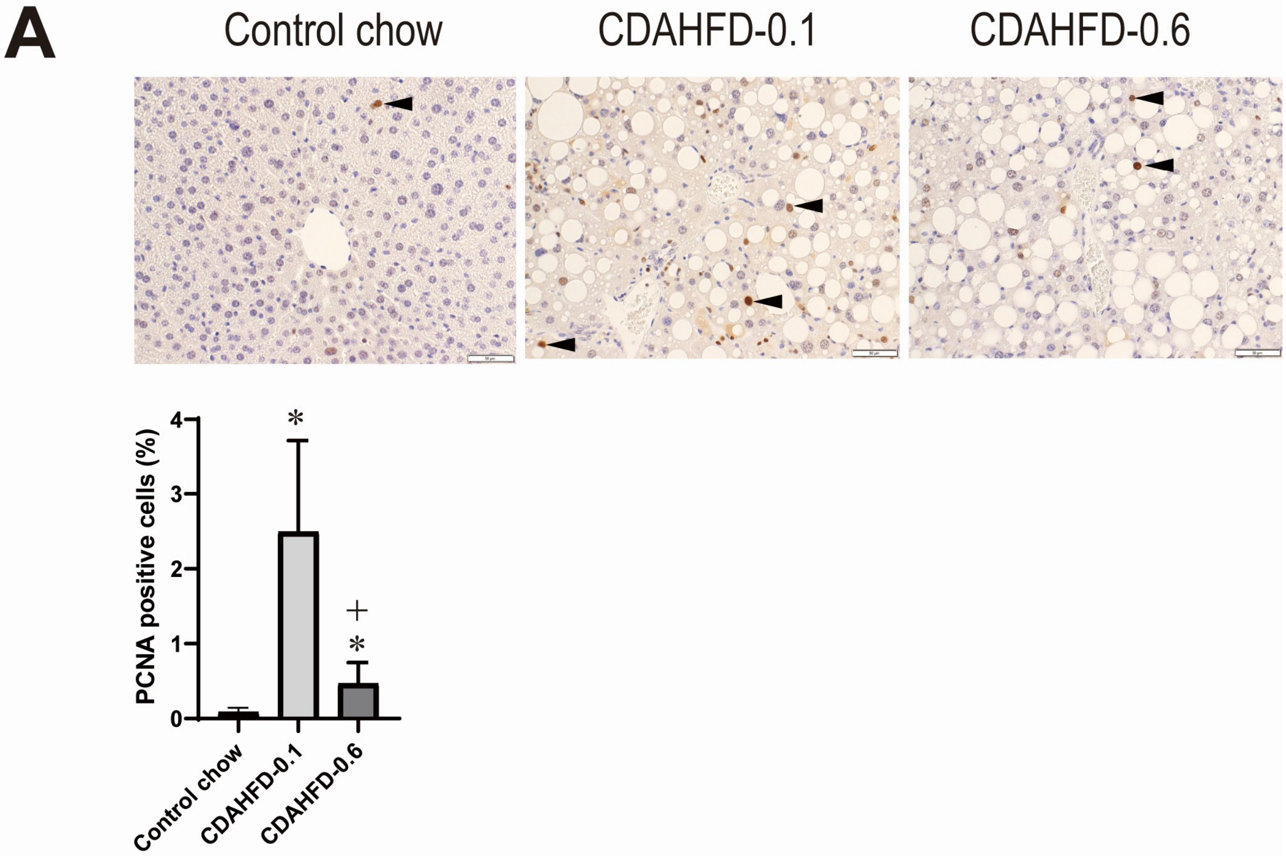

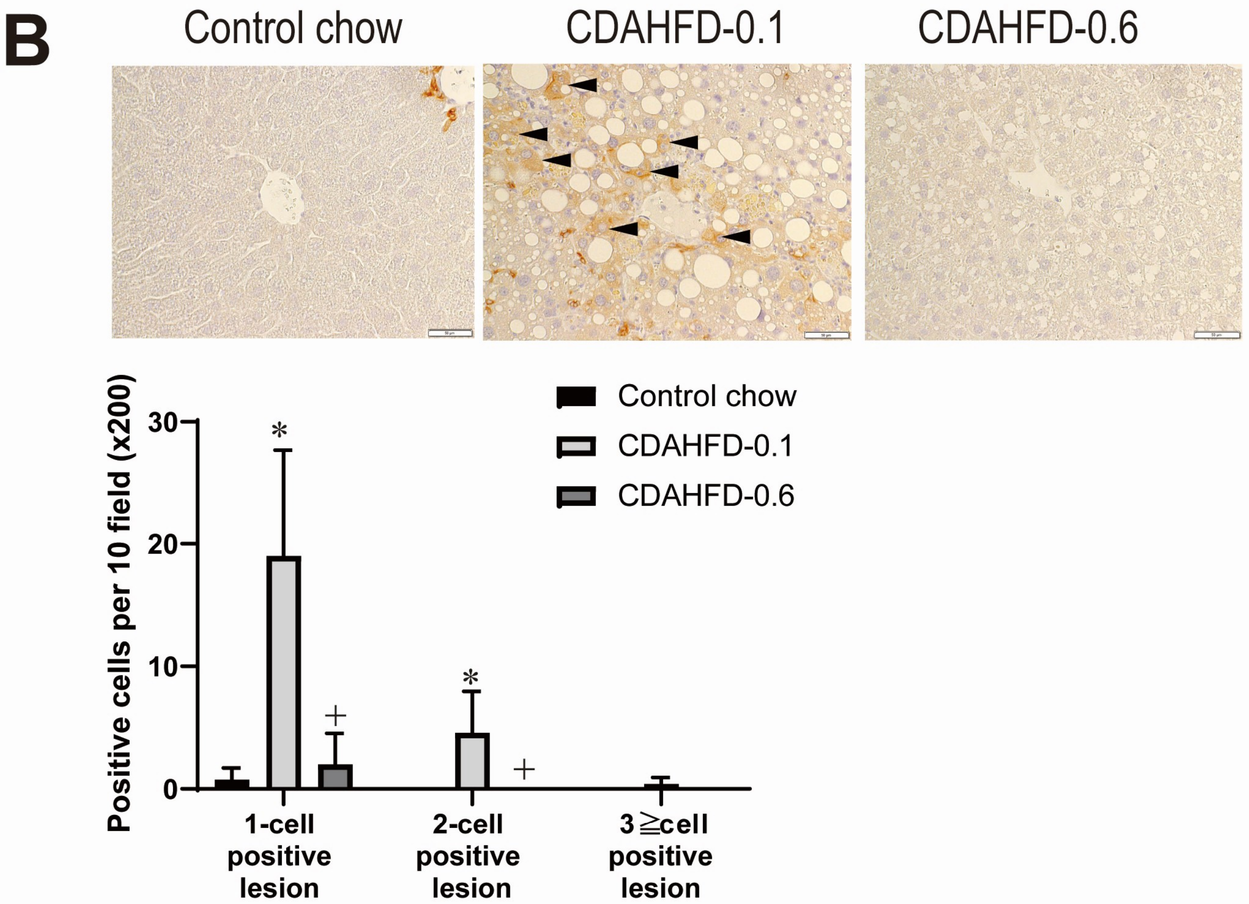

Supplemental Figure S4: Representative proliferative changes of the liver at the end of week 13.

The immunohistochemistry of PCNA (A) and CK8/18 (B). Arrows indicating positive cells.

*Significantly different from the control group value.

+Significantly different from the CDAHFD-0.1 group value.
