# Supplementary material for: Nonobese mice with nonalcoholic steatohepatitis fed on a choline‐deficient, l‐amino acid‐defined, high‐fat diet exhibit alterations in signaling pathways
Source: FEBS Open Bio. 2021 Sep 21;11(11):2950–65. doi: 10.1002/2211-5463.13272 (PMC8564345; doi:10.1002/2211-5463.13272)
Supplement: Supplementary file 5 — Table S1. Compositions of experimental diets used in this study. Table S2. Sequence information of primers for the qPCR analyses. [file FEB4-11-2950-s007.docx]

**Supplemental Table S1. Compositions of experimental diets used in this study.**

| **Ingredient (g/kg diet)** | **CDAHFD-0.1** | **CDAHFD-0.6** |
| --- | --- | --- |
| L-Cystine | 4.2 | 4.2 |
| L-Isoleucine | 7.6 | 7.6 |
| L-Leucine | 15.8 | 15.8 |
| L-Lysine | 13.2 | 13.2 |
| L-Methionine | 0.8 | 5.1 |
| L-Phenylalanine | 8.4 | 8.4 |
| L-Threonine | 7.2 | 7.2 |
| L-Tryptophan | 2.1 | 2.1 |
| L-Valine | 9.3 | 9.3 |
| L-Histidine | 4.6 | 4.6 |
| L-Alanine | 5.1 | 5.1 |
| L-Arginine | 6.0 | 6.0 |
| L-Aspartic Acid | 12.1 | 12.1 |
| L-Glutamic Acid | 38.2 | 38.2 |
| Glycine | 3.0 | 3.0 |
| L-Proline | 17.8 | 17.8 |
| L-Serine | 10.0 | 10.0 |
| L-Tyrosine | 9.2 | 9.2 |
| Corn Starch | 77.1 | 77.1 |
| Maltodextrin 10 | 100 | 100 |
| Sucrose | 172.8 | 172.8 |
| Cellulose | 50 | 50 |
| Soybean Oil | 25 | 25 |
| Lard | 177.5 | 177.5 |
| Mineral Mix S10026 | 10 | 10 |
| DiCalcium Phosphate | 13 | 13 |
| Calcium Carbonate | 5.5 | 5.5 |
| Potassium Citrate, 1 H2O | 16.5 | 16.5 |
| Sodium BiCarbonate | 7.5 | 7.5 |
| Vitamin Mix V10001 | 10 | 10 |
| Choline | 0 | 0 |
| Dye | 0.05 | 0.05 |
| Total | 839.55 | 839.75 |
|  |  |  |
| **% (w/w)** |  |  |
| Protein | 21 | 21 |
| Carbohydrate | 43 | 42 |
| Fat | 24 | 24 |
|  |  |  |
| **kcal %** |  |  |
| Protein | 18 | 18 |
| Carbohydrate | 36 | 36 |
| Fat | 46 | 46 |

**Supplemental Table S2. Sequence information of primers for the qPCR analyses**

| **Gene name** | **Forward primer**  **(5’ to 3’)** | **Reverse primer**  **(5’ to 3’)** |
| --- | --- | --- |
| TNFα | AGGGTCTGGGCCATAGAACT | CCACCACGCTCTTCTGTCTAC |
| CCR-2 | AGCACATGTGGTGAATCCAA | TGCCATCATAAAGGAGCCA |
| CD68 | ACCGCCATGTAGTCCAGGTA | ATCCCCACCTGTCTCTCTCA |
| p47phox | GATGTTCCCCATTGAGGCCG | GTTTCAGGTCATCAGGCCGC |
| P67phox | CTGGCTGAGGCCATCAGACT | AGGCCACTGCAGAGTGCTTG |
| TGFβ1 | GTGGAAATCAACGGGATCAG | ACTTCCAACCCAGGTCCTTC |
| Col1a1 | TAGGCCATTGTGTATGCAGC | ACATGTTCAGCTTTGTGGACC |
| Col4a1 | CACATTTTCCACAGCCAGAG | GTCTGGCTTCTGCTGCTCTT |
| TIMP1 | AGGTGGTCTCGTTGATTTCT | GTAAGGCCTGTAGCTGTGCC |
